# Synthesis and crystal structure of a Pd^II^ complex of *ortho*-xylylenebis(pyridyl­triazole)

**DOI:** 10.1107/S2414314623003620

**Published:** 2023-04-25

**Authors:** Uttam Pokharel, Aaron Naquin, Frank Fronczek

**Affiliations:** a Department of Chemistry and Physical Sciences, Nicholls State University, 906 E. 1st St., Thibodaux, Louisiana 70301, USA; b Department of Chemistry, Louisiana State University, Choppin Hall, Baton Rouge, Louisiana 70803, USA; Zhejiang University (Yuquan Campus), China

**Keywords:** *o*-xylylenebispyridyl­triazole, Pd^II^ complex, X-ray crystallography, metal–organic supra­molecules, square-planar geometry

## Abstract

A tetra­dentate ligand, namely, *ortho*-xylylenebis(pyridyl­triazole), *o*-xpt, was synthesized using the ‘click’ method and complexed with Pd(BF_4_)_2_. In the title com­plex, bis­{μ-1,1′-(*o*-xylylene)bis­[4-(pyridin-2-yl)triazole]-κ*N* ^3^:*N* ^3′^}dipalladi­um(II) tetra­kis­(tetrafluoridoborate)–di­methyl­formamide–diethyl ether (1/2/1), as the BF_4_ salt, and including di­methyl­formamide and diethyl ether solvent mol­ecules, with stoichiometry [Pd_2_(C_22_H_18_N_8_)_2_](BF_4_)_4_·2C_3_H_7_NO·C_4_H_10_O, the Pd complex and the disordered diethyl ether mol­ecule lie on inversion centers. The ligand coordinates to the Pd^II^ centers with square-planar geometry, forming a dimeric macrocycle. The Pd⋯Pd separation in the complex [Pd_2_(*o*-xpt)_2_]^4+^ cation is 3.6184 (4) Å. In the crystal, the complex mol­ecules are stacked along the *b* axis, with π–π inter­actions between the pyridyl­triazole ligands of two mol­ecules.

## Abstract 2

The *ortho*-xylylenebis(pyridyl­triazole) ligand coordinate with Pd^II^ giving dimeric square-planar complexes on which the Pd⋯Pd separation is 3.6184 (4) Å.

## Graphical abstract

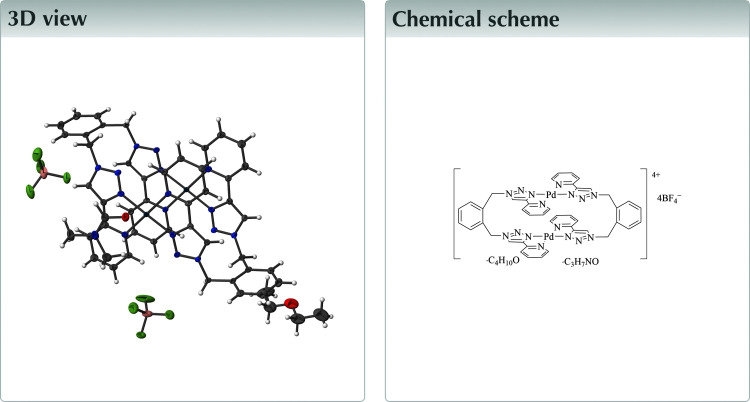

## Structure description

The self-assembly of polydentate ligands with transition-metal ions to create functional metal–organic supra­molecules has been of great inter­est in recent years. In particular, the complexation of 2-pyridyl-1,2,3-triazole chelating pockets with transition-metal ions has intensified due to the ease of synthesis of the ligands through the copper-catalyzed azide–alkyne cyclo­addition (CuAAC) reaction (Crowley & McMorran, 2012 ▸). We and others have studied the complexation of *ortho*-, *meta*-, and *para*-xylylene-bridged pyridyl­triazole tetra­dentate ligand with Cu^II^ (Pokharel *et al.*, 2013 ▸, 2014 ▸), Ni^II^ (Pokharel *et al.*, 2020*b* ▸), Fe^II^ (Vellas *et al.*, 2013 ▸), Ag^I^ (Gower & Crowley, 2010 ▸), and Cu^I^ (Zhao *et al.*, 2013 ▸). We have recently studied the crystal structure of 1,1-bis­(pyridyl­triazoylmeth­yl)ferrocene and its complexation with Cu^I^ (Pokharel *et al.*, 2020*a* ▸). As an extension of this work, we were also inter­ested in studying the complexation of *ortho*-xy­lyl­ene­bis(pyridyl­triazole), *o*-xpt, with Pd^II^. Herein, we report the synthesis and crystal structure of the title compound.

**Figure 1:**
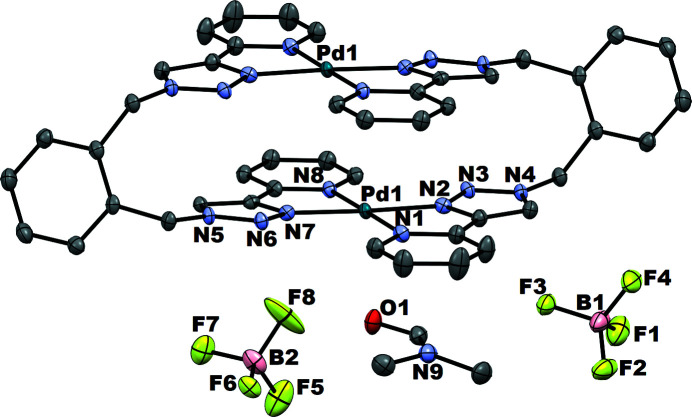
The mol­ecular structure of the title compound with the atom numbering. Displacement ellipsoids are drawn at the 50% probability level, and H atoms and disordered solvent mol­ecules have been omitted for clarity.

The structure of the title compound consists of the cationic Pd^II^ complex [Pd_2_(*o*-xpt)_2_]^4+^ lying on an inversion center, two BF_4_^−^ anions in general positions, a di­methyl­formamide solvent mol­ecule in a general position and a diethyl ether solvent mol­ecule disordered about an inversion center (Fig. 1 ▸). In the complex, two Pd^II^ cations are coordinated by two tetra­dentate *o*-xpt ligands, giving a dimeric macrocycle. The two pyridyl­triazole units are coordinated to each metal center in a *trans* fashion. The Pd^II^ centers are tetra­coordinated in a square-planar geometry defined by four (N1, N2, N7 and N8) atoms of two pyridyl­triazole moieties. The N—Pd—N chelating angles are N1—Pd1—N2 = 79.46 (8)° and N7—Pd1—N8 = 79.75 (8)°. The N(py)—Pd bonds [py is pyridine; average 2.055 (2) Å] are slightly longer than N(trz)—Pd bonds [trz is triazole; average 1.995 (2) Å], suggesting the triazolium N atom coordinates more strongly to the Pd center than the pyridyl N atom of the ligand. These values are typical for the reported mononuclear Pd^II^ complex of pyridyl­triazole ligands (Kilpin *et al.*, 2011 ▸). The Pd⋯Pd separation in the complex is 3.6184 (4) Å. Two phenyl­ene moieties in the complex are in an antiparallel orientation, with an inter­planar separation of 7.802 Å.

**Figure 2:**
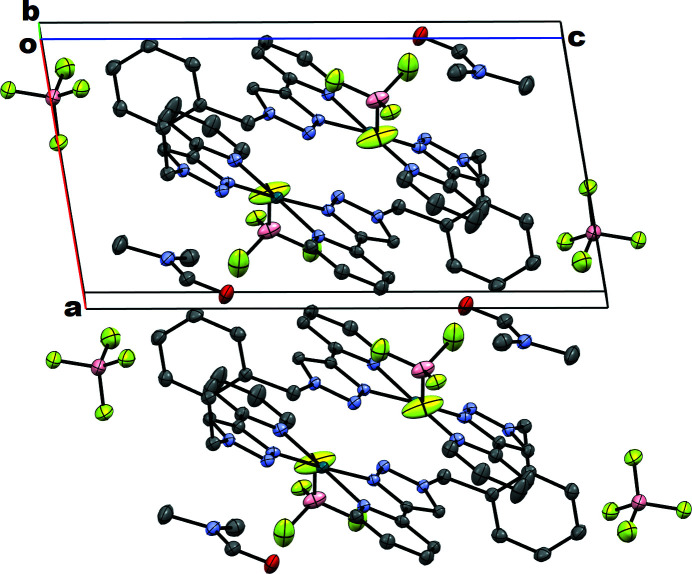
A view along the *b* axis of the crystal packing of the title compound. Disordered diethyl ether mol­ecules have been omitted for clarity.

In the crystal packing, the pyridyl­triazole units between two adjacent mol­ecules are associated in a head-to-tail arrangement (the electron-rich pyridyl group of one molecule stacks over the electron-deficient triazole group of the other molecule) with an average interplanar distance of 3.364 Å, indicating π–π interaction between the mol­ecules; this is shown along the *b* axis in Fig. 2 ▸.

## Synthesis and crystallization

To a stirred solution of [Pd(CH_3_CN)_4_]BF_4_ (0.112 mg, 0.253 mol) in aceto­nitrile (5 ml), *o*-xpt (0.100 g, 0.253 mmol) in aceto­nitrile (5 ml) was added dropwise. The solution was stirred for 1 h at room temperature. The volatiles were removed *in vacuo*. The residue was washed with di­chloro­methane (2 ml), followed by methanol (2 ml), and dried under vacuum to give [Pd_2_(*o*-xpt)_2_](BF_4_)_4_ (0.126 mg, 74%) as a pale-yellow solid. Crystals suitable for X-ray analysis were obtained by slow vapor diffusion of diethyl ether into a di­methyl­formamide (DMF) solution of the complex at room tem­per­ature. Our attempts to obtain a clean ^1^H NMR spectrum in DMSO-*d*_6_ were not successful, possibly due to the labile nature of the complex in solution. High resolution ESI–MS analysis showed a monocationic signal at *m*/*z* 1255.1478 {calculated 1255.1540 for [Pd_2_(*o*-xpt)_2_(BF_4_)_4_]^+^}.

## Refinement

Crystal data, data collection and structure refinement details are summarized in Table 1 ▸. All H atoms were located in difference maps and thereafter treated as riding in geometrically idealized positions, with C—H = 0.95 Å for C*sp* ^2^, 0.98 Å for methyl, and 0.99 Å for CH_2_. *U*_iso_(H) values were assigned as 1.2*U*_eq_ for the attached atom (1.5 for meth­yl). A torsional parameter was refined for each methyl group, except for those of the diethyl ether mol­ecule, which were staggered with respect to CH_2_. The diethyl ether solvent mol­ecule is disordered about an inversion center with two half-populated sites. A number of distance and displacement parameter restraints were necessary to model the disorder.

## Supplementary Material

Crystal structure: contains datablock(s) I, glonal. DOI: 10.1107/S2414314623003620/xu4050sup1.cif

Structure factors: contains datablock(s) I. DOI: 10.1107/S2414314623003620/xu4050Isup2.hkl

CCDC reference: 2257865

Additional supporting information:  crystallographic information; 3D view; checkCIF report

## Figures and Tables

**Table 1:** Experimental details

Crystal data	
Chemical formula	[Pd_2_(C_22_H_18_N_8_)_2_](BF_4_)_4_·2C_3_H_7_NO·C_4_H_10_O
*M*_r_	1569.24
Crystal system, space group	Triclinic, *P* 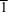
Temperature (K)	90
*a*, *b*, *c* (Å)	8.3546 (4), 12.4468 (5), 15.4504 (6)
α, β, γ (°)	85.540 (2), 79.660 (2), 76.997 (2)
*V* (Å^3^)	1538.86 (11)
*Z*	1
Radiation type	Cu *K*α
μ (mm^−1^)	5.68
Crystal size (mm)	0.08 × 0.04 × 0.01

Data collection	
Diffractometer	Bruker Kappa APEXII CCD DUO
Absorption correction	Multi-scan (*SADABS*; Krause *et al.*, 2015 ▸)
*T*_min_, *T*_max_	0.765, 0.945
No. of measured, independent and observed [*I* > 2σ(*I*)] reflections	17544, 5427, 4983
*R*_int_	0.039
(sin θ/λ)_max_ (Å^−1^)	0.603

Refinement	
*R*[*F* ^2^ > 2σ(*F* ^2^)], *wR*(*F* ^2^), *S*	0.029, 0.076, 1.03
No. of reflections	5427
No. of parameters	456
No. of restraints	34
H-atom treatment	H-atom parameters constrained
Δρ_max_, Δρ_min_ (e Å^−3^)	0.68, −0.41

Computer programs: *APEX2* and *SAINT* (Bruker, 2016 ▸), *SHELXT2014* (Sheldrick, 2015*a* ▸), *SHELXL2017* (Sheldrick, 2015*b* ▸), *Mercury* (Macrae *et al.*, 2020 ▸), and *publCIF* (Westrip, 2010 ▸).

## full crystallographic data

### Crystal data

**Table:**

[Pd_2_(C_22_H_18_N_8_)_2_](BF_4_)_4_·2C_3_H_7_NO·C_4_H_10_O	*Z* = 1
*M**_r_* = 1569.24	*F*(000) = 790
Triclinic, *P*1	*D*_x_ = 1.693 Mg m^−^^3^
*a* = 8.3546 (4) Å	Cu *K*α radiation, λ = 1.54184 Å
*b* = 12.4468 (5) Å	Cell parameters from 7868 reflections
*c* = 15.4504 (6) Å	θ = 2.9–68.3°
α = 85.540 (2)°	µ = 5.68 mm^−^^1^
β = 79.660 (2)°	*T* = 90 K
γ = 76.997 (2)°	Fragment, pale yellow
*V* = 1538.86 (11) Å^3^	0.08 × 0.04 × 0.01 mm

### Data collection

**Table:**

Bruker Kappa APEXII CCD DUO diffractometer	5427 independent reflections
Radiation source: IµS microfocus	4983 reflections with *I* > 2σ(*I*)
QUAZAR multilayer optics monochromator	*R*_int_ = 0.039
φ and ω scans	θ_max_ = 68.5°, θ_min_ = 3.7°
Absorption correction: multi-scan (SADABS; Krause *et al.*, 2015)	*h* = −9→10
*T*_min_ = 0.765, *T*_max_ = 0.945	*k* = −14→10
17544 measured reflections	*l* = −18→18

### Refinement

**Table:**

Refinement on *F*^2^	34 restraints
Least-squares matrix: full	Hydrogen site location: inferred from neighbouring sites
*R*[*F*^2^ > 2σ(*F*^2^)] = 0.029	H-atom parameters constrained
*wR*(*F*^2^) = 0.076	*w* = 1/[σ^2^(*F*_o_^2^) + (0.0428*P*)^2^ + 1.022*P*] where *P* = (*F*_o_^2^ + 2*F*_c_^2^)/3
*S* = 1.03	(Δ/σ)_max_ = 0.001
5427 reflections	Δρ_max_ = 0.68 e Å^−^^3^
456 parameters	Δρ_min_ = −0.41 e Å^−^^3^

### Special details

**Table:**

Geometry. All e.s.d.'s (except the e.s.d. in the dihedral angle between two l.s. planes) are estimated using the full covariance matrix. The cell e.s.d.'s are taken into account individually in the estimation of e.s.d.'s in distances, angles and torsion angles; correlations between e.s.d.'s in cell parameters are only used when they are defined by crystal symmetry. An approximate (isotropic) treatment of cell e.s.d.'s is used for estimating e.s.d.'s involving l.s. planes.

### Fractional atomic coordinates and isotropic or equivalent isotropic displacement parameters (Å^2^)

**Table:**

	*x*	*y*	*z*	*U*_iso_*/*U*_eq_	Occ. (<1)
Pd1	0.62296 (2)	0.54481 (2)	0.40085 (2)	0.01235 (8)	
N1	0.4910 (3)	0.67202 (18)	0.33215 (15)	0.0177 (5)	
N2	0.5621 (3)	0.46128 (17)	0.31114 (14)	0.0154 (4)	
N3	0.5861 (3)	0.35595 (17)	0.29813 (14)	0.0154 (4)	
N4	0.5089 (3)	0.34974 (17)	0.23042 (14)	0.0156 (4)	
N5	0.2946 (3)	0.26548 (17)	0.41317 (14)	0.0145 (4)	
N6	0.3668 (3)	0.26845 (17)	0.48321 (14)	0.0157 (4)	
N7	0.3247 (3)	0.37183 (17)	0.50545 (14)	0.0144 (4)	
N8	0.2416 (3)	0.58271 (17)	0.53220 (14)	0.0152 (4)	
C1	0.4641 (4)	0.7791 (2)	0.34815 (19)	0.0218 (6)	
H1	0.506819	0.800711	0.395483	0.026*	
C2	0.3757 (4)	0.8589 (2)	0.2975 (2)	0.0310 (7)	
H2	0.358914	0.934618	0.309918	0.037*	
C3	0.3116 (5)	0.8291 (3)	0.2290 (2)	0.0378 (8)	
H3	0.248716	0.883448	0.194498	0.045*	
C4	0.3410 (4)	0.7178 (2)	0.2113 (2)	0.0294 (7)	
H4	0.299455	0.694820	0.164041	0.035*	
C5	0.4312 (3)	0.6413 (2)	0.26347 (18)	0.0189 (5)	
C6	0.4704 (3)	0.5227 (2)	0.25314 (17)	0.0173 (5)	
C7	0.4363 (3)	0.4500 (2)	0.20031 (17)	0.0175 (5)	
H7	0.375086	0.466634	0.152977	0.021*	
C8	0.5148 (3)	0.2393 (2)	0.19970 (18)	0.0176 (5)	
H8A	0.580939	0.182775	0.235508	0.021*	
H8B	0.572049	0.233842	0.137765	0.021*	
C9	0.3437 (3)	0.2151 (2)	0.20577 (17)	0.0171 (5)	
C10	0.2720 (4)	0.2300 (2)	0.12980 (18)	0.0212 (6)	
H10	0.329220	0.257654	0.077049	0.025*	
C11	0.1185 (4)	0.2050 (2)	0.12995 (19)	0.0255 (6)	
H11	0.070824	0.216010	0.077702	0.031*	
C12	0.0348 (4)	0.1642 (2)	0.20627 (19)	0.0252 (6)	
H12	−0.069897	0.146220	0.206446	0.030*	
C13	0.1041 (3)	0.1494 (2)	0.28270 (18)	0.0206 (6)	
H13	0.046137	0.121030	0.334933	0.025*	
C14	0.2570 (3)	0.1754 (2)	0.28398 (17)	0.0161 (5)	
C15	0.3232 (3)	0.1588 (2)	0.36990 (17)	0.0171 (5)	
H15A	0.266954	0.107126	0.409490	0.021*	
H15B	0.444077	0.125712	0.358676	0.021*	
C16	0.2068 (3)	0.3655 (2)	0.39066 (16)	0.0146 (5)	
H16	0.144232	0.383836	0.343930	0.018*	
C17	0.2283 (3)	0.4345 (2)	0.45040 (16)	0.0148 (5)	
C18	0.1818 (3)	0.5523 (2)	0.46399 (17)	0.0154 (5)	
C19	0.0907 (3)	0.6280 (2)	0.41110 (17)	0.0176 (5)	
H19	0.049619	0.604355	0.364009	0.021*	
C20	0.0605 (3)	0.7394 (2)	0.42825 (18)	0.0198 (6)	
H20	−0.000657	0.793352	0.392719	0.024*	
C21	0.1214 (3)	0.7704 (2)	0.49825 (18)	0.0198 (5)	
H21	0.102035	0.846091	0.511181	0.024*	
C22	0.2102 (3)	0.6906 (2)	0.54896 (17)	0.0178 (5)	
H22	0.250380	0.712569	0.597095	0.021*	
B1	0.7437 (5)	0.3502 (4)	−0.0041 (2)	0.0341 (8)	
F1	0.8450 (3)	0.2509 (2)	−0.03601 (14)	0.0497 (5)	
F2	0.7818 (2)	0.43622 (19)	−0.06116 (12)	0.0442 (5)	
F3	0.7724 (2)	0.36416 (16)	0.07958 (11)	0.0324 (4)	
F4	0.5762 (2)	0.34747 (19)	−0.00012 (12)	0.0429 (5)	
B2	0.7718 (4)	0.9939 (3)	0.3743 (3)	0.0305 (8)	
F5	0.8893 (3)	0.94186 (17)	0.30537 (14)	0.0536 (6)	
F6	0.7324 (2)	1.10683 (13)	0.35316 (12)	0.0273 (4)	
F7	0.8434 (3)	0.97815 (15)	0.45036 (12)	0.0394 (5)	
F8	0.6308 (3)	0.95332 (18)	0.3871 (2)	0.0773 (9)	
O1	0.9776 (3)	0.49882 (17)	0.27263 (13)	0.0280 (5)	
N9	0.8519 (3)	0.6141 (2)	0.17162 (16)	0.0248 (5)	
C23	0.9211 (3)	0.5157 (2)	0.20272 (19)	0.0239 (6)	
H23	0.927928	0.453656	0.169054	0.029*	
C24	0.8557 (4)	0.7145 (3)	0.2135 (2)	0.0314 (7)	
H24A	0.857946	0.698513	0.276396	0.047*	
H24B	0.756191	0.771129	0.205856	0.047*	
H24C	0.955517	0.741219	0.186200	0.047*	
C25	0.8014 (4)	0.6276 (3)	0.0849 (2)	0.0364 (8)	
H25A	0.876235	0.665880	0.044140	0.055*	
H25B	0.686950	0.671119	0.089651	0.055*	
H25C	0.807063	0.554943	0.062787	0.055*	
O2	0.4535 (11)	0.9884 (7)	1.0054 (7)	0.067 (3)	0.5
C26	0.3717 (19)	1.0268 (16)	0.9327 (11)	0.072 (4)	0.5
H26A	0.441392	0.995410	0.877955	0.086*	0.5
H26B	0.353021	1.108211	0.926463	0.086*	0.5
C27	0.2032 (17)	0.9910 (10)	0.9481 (10)	0.094 (4)	0.5
H27A	0.145357	1.017146	0.898066	0.141*	0.5
H27B	0.222622	0.910310	0.953778	0.141*	0.5
H27C	0.134518	1.022771	1.002139	0.141*	0.5
C28	0.6073 (17)	1.0284 (14)	0.9992 (12)	0.067 (3)	0.5
H28A	0.580069	1.110043	0.999418	0.081*	0.5
H28B	0.680609	1.006214	0.942838	0.081*	0.5
C29	0.699 (2)	0.9832 (15)	1.0748 (12)	0.086 (5)	0.5
H29A	0.801333	1.011668	1.068363	0.129*	0.5
H29B	0.628024	1.006203	1.130644	0.129*	0.5
H29C	0.728516	0.902423	1.074091	0.129*	0.5

### Atomic displacement parameters (Å^2^)

**Table:**

	*U*^11^	*U*^22^	*U*^33^	*U*^12^	*U*^13^	*U*^23^
Pd1	0.01319 (11)	0.00994 (11)	0.01434 (11)	−0.00127 (7)	−0.00446 (7)	−0.00152 (7)
N1	0.0181 (11)	0.0147 (11)	0.0203 (11)	−0.0029 (9)	−0.0039 (9)	−0.0016 (9)
N2	0.0137 (10)	0.0129 (10)	0.0187 (11)	−0.0014 (8)	−0.0024 (9)	−0.0003 (8)
N3	0.0146 (10)	0.0140 (10)	0.0183 (11)	−0.0027 (8)	−0.0038 (9)	−0.0033 (8)
N4	0.0164 (11)	0.0157 (11)	0.0156 (10)	−0.0035 (9)	−0.0041 (9)	−0.0039 (8)
N5	0.0162 (11)	0.0131 (10)	0.0154 (10)	−0.0024 (8)	−0.0052 (8)	−0.0034 (8)
N6	0.0168 (11)	0.0130 (10)	0.0181 (11)	−0.0023 (8)	−0.0055 (9)	−0.0023 (8)
N7	0.0144 (10)	0.0134 (10)	0.0150 (10)	−0.0019 (8)	−0.0024 (8)	−0.0006 (8)
N8	0.0155 (11)	0.0145 (10)	0.0161 (10)	−0.0026 (8)	−0.0030 (9)	−0.0035 (8)
C1	0.0275 (15)	0.0146 (13)	0.0239 (14)	−0.0032 (11)	−0.0067 (12)	−0.0026 (11)
C2	0.0466 (19)	0.0138 (13)	0.0343 (17)	−0.0009 (13)	−0.0178 (15)	−0.0021 (12)
C3	0.059 (2)	0.0178 (15)	0.0375 (18)	0.0055 (14)	−0.0276 (17)	−0.0004 (13)
C4	0.0412 (18)	0.0213 (14)	0.0272 (16)	0.0004 (13)	−0.0188 (14)	−0.0006 (12)
C5	0.0213 (13)	0.0167 (13)	0.0187 (13)	−0.0022 (11)	−0.0056 (11)	−0.0014 (10)
C6	0.0177 (13)	0.0168 (13)	0.0174 (13)	−0.0028 (10)	−0.0048 (10)	0.0002 (10)
C7	0.0192 (13)	0.0175 (13)	0.0160 (12)	−0.0027 (10)	−0.0049 (10)	−0.0020 (10)
C8	0.0187 (13)	0.0141 (12)	0.0205 (13)	−0.0021 (10)	−0.0044 (11)	−0.0045 (10)
C9	0.0178 (13)	0.0129 (12)	0.0208 (13)	−0.0013 (10)	−0.0043 (10)	−0.0050 (10)
C10	0.0257 (14)	0.0183 (13)	0.0200 (13)	−0.0042 (11)	−0.0048 (11)	−0.0027 (10)
C11	0.0239 (15)	0.0297 (15)	0.0252 (15)	−0.0044 (12)	−0.0107 (12)	−0.0032 (12)
C12	0.0189 (14)	0.0300 (15)	0.0298 (15)	−0.0071 (12)	−0.0073 (12)	−0.0061 (12)
C13	0.0198 (13)	0.0194 (13)	0.0226 (14)	−0.0040 (11)	−0.0019 (11)	−0.0049 (11)
C14	0.0187 (13)	0.0112 (11)	0.0180 (13)	0.0009 (10)	−0.0055 (10)	−0.0056 (9)
C15	0.0224 (13)	0.0109 (12)	0.0182 (13)	−0.0014 (10)	−0.0055 (11)	−0.0030 (10)
C16	0.0143 (12)	0.0143 (12)	0.0154 (12)	−0.0024 (10)	−0.0034 (10)	−0.0009 (9)
C17	0.0128 (12)	0.0150 (12)	0.0150 (12)	−0.0010 (10)	−0.0009 (10)	−0.0002 (9)
C18	0.0148 (12)	0.0118 (12)	0.0185 (13)	−0.0007 (10)	−0.0010 (10)	−0.0045 (10)
C19	0.0170 (13)	0.0187 (13)	0.0169 (13)	−0.0011 (10)	−0.0045 (10)	−0.0024 (10)
C20	0.0224 (14)	0.0150 (13)	0.0209 (13)	0.0009 (11)	−0.0068 (11)	−0.0009 (10)
C21	0.0232 (14)	0.0139 (12)	0.0217 (14)	−0.0015 (10)	−0.0046 (11)	−0.0018 (10)
C22	0.0180 (13)	0.0162 (13)	0.0182 (13)	−0.0015 (10)	−0.0017 (10)	−0.0044 (10)
B1	0.0245 (18)	0.057 (2)	0.0206 (17)	−0.0070 (16)	−0.0061 (14)	0.0010 (16)
F1	0.0433 (12)	0.0655 (14)	0.0374 (11)	−0.0002 (10)	−0.0085 (9)	−0.0133 (10)
F2	0.0301 (10)	0.0731 (15)	0.0282 (10)	−0.0117 (10)	−0.0081 (8)	0.0160 (9)
F3	0.0284 (9)	0.0488 (11)	0.0214 (9)	−0.0099 (8)	−0.0073 (7)	0.0014 (8)
F4	0.0256 (10)	0.0762 (15)	0.0305 (10)	−0.0159 (9)	−0.0090 (8)	0.0007 (9)
B2	0.0278 (18)	0.0158 (15)	0.050 (2)	−0.0031 (13)	−0.0145 (16)	0.0013 (14)
F5	0.0783 (16)	0.0320 (11)	0.0404 (11)	0.0168 (10)	−0.0169 (11)	−0.0069 (9)
F6	0.0243 (8)	0.0179 (8)	0.0408 (10)	−0.0040 (7)	−0.0110 (7)	0.0029 (7)
F7	0.0525 (12)	0.0270 (9)	0.0368 (10)	0.0019 (8)	−0.0169 (9)	0.0014 (8)
F8	0.0483 (14)	0.0306 (11)	0.166 (3)	−0.0230 (10)	−0.0454 (17)	0.0217 (15)
O1	0.0344 (12)	0.0262 (11)	0.0247 (11)	−0.0014 (9)	−0.0149 (9)	−0.0006 (8)
N9	0.0210 (12)	0.0313 (13)	0.0234 (12)	−0.0070 (10)	−0.0079 (10)	0.0047 (10)
C23	0.0200 (14)	0.0287 (15)	0.0234 (14)	−0.0045 (12)	−0.0060 (11)	0.0003 (12)
C24	0.0302 (16)	0.0278 (16)	0.0364 (17)	−0.0059 (13)	−0.0081 (14)	0.0030 (13)
C25	0.0362 (18)	0.050 (2)	0.0264 (16)	−0.0138 (15)	−0.0149 (14)	0.0119 (14)
O2	0.077 (7)	0.041 (4)	0.071 (3)	−0.004 (5)	0.013 (5)	−0.006 (3)
C26	0.091 (9)	0.048 (6)	0.067 (7)	0.000 (6)	−0.007 (6)	−0.004 (5)
C27	0.100 (9)	0.057 (7)	0.121 (11)	−0.028 (6)	0.021 (8)	−0.038 (7)
C28	0.077 (7)	0.041 (4)	0.071 (3)	−0.004 (5)	0.013 (5)	−0.006 (3)
C29	0.107 (13)	0.047 (8)	0.082 (9)	0.011 (9)	0.007 (10)	0.002 (6)

### Geometric parameters (Å, º)

**Table:**

Pd1—N2	1.994 (2)	C16—C17	1.369 (4)
Pd1—N7^i^	2.007 (2)	C16—H16	0.9500
Pd1—N1	2.055 (2)	C17—C18	1.451 (4)
Pd1—N8^i^	2.056 (2)	C18—C19	1.384 (4)
N1—C1	1.336 (4)	C19—C20	1.390 (4)
N1—C5	1.364 (3)	C19—H19	0.9500
N2—N3	1.307 (3)	C20—C21	1.389 (4)
N2—C6	1.360 (3)	C20—H20	0.9500
N3—N4	1.339 (3)	C21—C22	1.381 (4)
N4—C7	1.346 (3)	C21—H21	0.9500
N4—C8	1.477 (3)	C22—H22	0.9500
N5—N6	1.337 (3)	B1—F3	1.387 (4)
N5—C16	1.350 (3)	B1—F2	1.389 (4)
N5—C15	1.485 (3)	B1—F4	1.398 (4)
N6—N7	1.311 (3)	B1—F1	1.400 (5)
N7—C17	1.357 (3)	B2—F8	1.360 (4)
N8—C22	1.346 (3)	B2—F7	1.394 (4)
N8—C18	1.356 (3)	B2—F6	1.396 (4)
C1—C2	1.379 (4)	B2—F5	1.401 (5)
C1—H1	0.9500	O1—C23	1.240 (3)
C2—C3	1.379 (4)	N9—C23	1.325 (4)
C2—H2	0.9500	N9—C24	1.459 (4)
C3—C4	1.392 (4)	N9—C25	1.462 (4)
C3—H3	0.9500	C23—H23	0.9500
C4—C5	1.378 (4)	C24—H24A	0.9800
C4—H4	0.9500	C24—H24B	0.9800
C5—C6	1.453 (4)	C24—H24C	0.9800
C6—C7	1.372 (4)	C25—H25A	0.9800
C7—H7	0.9500	C25—H25B	0.9800
C8—C9	1.512 (4)	C25—H25C	0.9800
C8—H8A	0.9900	O2—C26	1.415 (14)
C8—H8B	0.9900	O2—C28	1.465 (12)
C9—C10	1.393 (4)	C26—C27	1.543 (14)
C9—C14	1.408 (4)	C26—H26A	0.9900
C10—C11	1.385 (4)	C26—H26B	0.9900
C10—H10	0.9500	C27—H27A	0.9800
C11—C12	1.381 (4)	C27—H27B	0.9800
C11—H11	0.9500	C27—H27C	0.9800
C12—C13	1.388 (4)	C28—C29	1.515 (16)
C12—H12	0.9500	C28—H28A	0.9900
C13—C14	1.390 (4)	C28—H28B	0.9900
C13—H13	0.9500	C29—H29A	0.9800
C14—C15	1.510 (3)	C29—H29B	0.9800
C15—H15A	0.9900	C29—H29C	0.9800
C15—H15B	0.9900		
			
N2—Pd1—N7^i^	177.57 (8)	N5—C16—C17	104.4 (2)
N2—Pd1—N1	79.46 (8)	N5—C16—H16	127.8
N7^i^—Pd1—N1	100.55 (8)	C17—C16—H16	127.8
N2—Pd1—N8^i^	100.27 (8)	N7—C17—C16	107.1 (2)
N7^i^—Pd1—N8^i^	79.75 (8)	N7—C17—C18	116.8 (2)
N1—Pd1—N8^i^	179.01 (9)	C16—C17—C18	136.0 (2)
C1—N1—C5	119.1 (2)	N8—C18—C19	122.4 (2)
C1—N1—Pd1	125.63 (18)	N8—C18—C17	113.5 (2)
C5—N1—Pd1	115.24 (17)	C19—C18—C17	124.0 (2)
N3—N2—C6	111.1 (2)	C18—C19—C20	118.7 (2)
N3—N2—Pd1	132.75 (17)	C18—C19—H19	120.7
C6—N2—Pd1	116.02 (17)	C20—C19—H19	120.7
N2—N3—N4	105.32 (19)	C21—C20—C19	118.8 (2)
N3—N4—C7	112.2 (2)	C21—C20—H20	120.6
N3—N4—C8	118.0 (2)	C19—C20—H20	120.6
C7—N4—C8	129.8 (2)	C22—C21—C20	119.6 (2)
N6—N5—C16	112.4 (2)	C22—C21—H21	120.2
N6—N5—C15	118.5 (2)	C20—C21—H21	120.2
C16—N5—C15	129.2 (2)	N8—C22—C21	121.9 (2)
N7—N6—N5	105.12 (19)	N8—C22—H22	119.0
N6—N7—C17	111.1 (2)	C21—C22—H22	119.0
N6—N7—Pd1^i^	134.09 (16)	F3—B1—F2	110.5 (3)
C17—N7—Pd1^i^	114.81 (17)	F3—B1—F4	109.4 (3)
C22—N8—C18	118.5 (2)	F2—B1—F4	109.3 (3)
C22—N8—Pd1^i^	126.47 (17)	F3—B1—F1	109.2 (3)
C18—N8—Pd1^i^	115.02 (16)	F2—B1—F1	108.6 (3)
N1—C1—C2	121.5 (3)	F4—B1—F1	109.7 (3)
N1—C1—H1	119.2	F8—B2—F7	110.6 (3)
C2—C1—H1	119.2	F8—B2—F6	109.4 (3)
C3—C2—C1	120.1 (3)	F7—B2—F6	108.7 (3)
C3—C2—H2	120.0	F8—B2—F5	111.1 (3)
C1—C2—H2	120.0	F7—B2—F5	108.4 (3)
C2—C3—C4	118.7 (3)	F6—B2—F5	108.5 (3)
C2—C3—H3	120.6	C23—N9—C24	120.9 (2)
C4—C3—H3	120.6	C23—N9—C25	121.0 (3)
C5—C4—C3	118.9 (3)	C24—N9—C25	116.9 (3)
C5—C4—H4	120.6	O1—C23—N9	124.7 (3)
C3—C4—H4	120.6	O1—C23—H23	117.7
N1—C5—C4	121.7 (2)	N9—C23—H23	117.7
N1—C5—C6	113.4 (2)	N9—C24—H24A	109.5
C4—C5—C6	124.9 (3)	N9—C24—H24B	109.5
N2—C6—C7	106.7 (2)	H24A—C24—H24B	109.5
N2—C6—C5	115.9 (2)	N9—C24—H24C	109.5
C7—C6—C5	137.4 (2)	H24A—C24—H24C	109.5
N4—C7—C6	104.7 (2)	H24B—C24—H24C	109.5
N4—C7—H7	127.7	N9—C25—H25A	109.5
C6—C7—H7	127.7	N9—C25—H25B	109.5
N4—C8—C9	112.7 (2)	H25A—C25—H25B	109.5
N4—C8—H8A	109.1	N9—C25—H25C	109.5
C9—C8—H8A	109.1	H25A—C25—H25C	109.5
N4—C8—H8B	109.1	H25B—C25—H25C	109.5
C9—C8—H8B	109.1	C26—O2—C28	111.7 (9)
H8A—C8—H8B	107.8	O2—C26—C27	108.8 (12)
C10—C9—C14	119.1 (2)	O2—C26—H26A	109.9
C10—C9—C8	118.1 (2)	C27—C26—H26A	109.9
C14—C9—C8	122.7 (2)	O2—C26—H26B	109.9
C11—C10—C9	121.0 (3)	C27—C26—H26B	109.9
C11—C10—H10	119.5	H26A—C26—H26B	108.3
C9—C10—H10	119.5	C26—C27—H27A	109.5
C12—C11—C10	119.8 (3)	C26—C27—H27B	109.5
C12—C11—H11	120.1	H27A—C27—H27B	109.5
C10—C11—H11	120.1	C26—C27—H27C	109.5
C11—C12—C13	119.9 (3)	H27A—C27—H27C	109.5
C11—C12—H12	120.1	H27B—C27—H27C	109.5
C13—C12—H12	120.1	O2—C28—C29	111.9 (12)
C12—C13—C14	121.0 (3)	O2—C28—H28A	109.2
C12—C13—H13	119.5	C29—C28—H28A	109.2
C14—C13—H13	119.5	O2—C28—H28B	109.2
C13—C14—C9	119.1 (2)	C29—C28—H28B	109.2
C13—C14—C15	118.0 (2)	H28A—C28—H28B	107.9
C9—C14—C15	122.9 (2)	C28—C29—H29A	109.5
N5—C15—C14	110.7 (2)	C28—C29—H29B	109.5
N5—C15—H15A	109.5	H29A—C29—H29B	109.5
C14—C15—H15A	109.5	C28—C29—H29C	109.5
N5—C15—H15B	109.5	H29A—C29—H29C	109.5
C14—C15—H15B	109.5	H29B—C29—H29C	109.5
H15A—C15—H15B	108.1		

Symmetry code: (i) −*x*+1, −*y*+1, −*z*+1.
